# Development of Cloned B Cell Populations as Immunotherapeutic Agents Against MYC Carcinogenic Proteins

**DOI:** 10.34172/apb.46527

**Published:** 2026-06-27

**Authors:** Sushrut Joshi, Chitrali Talele, Gunosindhu Chakraborthy, Manisha Lalan, Sachin Mali

**Affiliations:** ^1^Department of Pharmaceutics, Parul Institute of Pharmacy & Research, Faculty of Pharmacy, Parul University, Vadodara, India; ^2^Department of Pharmacy, Sumandeep Vidyapeeth Deemed to be University, Vadodara, India; ^3^Department of Pharmaceutics, Bharati Vidyapeeth College of Pharmacy, Kolhapur, India

**Keywords:** MYC oncogene, Cancer immunotherapy, B-cells in tumor microenvironment, MYC-targeted therapy, Antibody engineering, CAR-B-cells, Synthetic lethality, Translational oncology

## Abstract

The MYC proto-oncogene is one of the most extensively dysregulated transcription factors in cancer, governing cell cycle progression, metabolic adaptation, and immune evasion. Despite its central role in tumor biology, MYC has been considered an undruggable target due to its nuclear localization, lack of enzymatic activity, and involvement in transcriptional regulation. The emergence of B-cell based immunotherapies has reshaped the understanding of tumor immunology, revealing that B cells exert both effector and regulatory roles in tumor microenvironment. The intersection between MYC biology and B-cell function is relevant, as MYC is indispensable for B-cell maturation and is frequently dysregulated in B-cell-derived malignancies such as Burkitt lymphoma and diffuse large B-cell lymphoma. We focus specifically on the intersection between MYC-driven oncogenic signaling and emerging B-cell-based immunotherapeutic platforms. This review proposes a conceptual reference framework in which MYC is considered not only as a proliferative driver but also as an immune-modulating oncogene that can aid in the design of humoral therapeutic strategies. Therapeutic strategies to inhibit MYC are systematically discussed, including BET and CDK inhibitors, disruption of MYC–MAX dimerization, RNA-based technologies, synthetic lethality approaches, and emerging PROTACs. Cloned B-cell populations, effector B-cells secreting intrabodies, CAR-B platforms, and antibody-based intracellular targeting are areas of focus. An overview is presented of preclinical and clinical advances made thus far, as well as challenges to the successful translation of these therapies for use in patients including tumor heterogeneity, limitations associated with delivery of agents, and risk of inducing autoimmunity. Forecasting the future provides rationale for combining MYC-targeting agents with immunotherapies and using personalized neoantigen-driven B-cell immunotherapies to treat patients with cancer. Overall, these findings support the concept that MYC B-cell-directed strategies will represent a novel means of treating patients with cancer through their development into new classes of therapeutics.

## Introduction

 Cancer continues to be a global health concern, with an ongoing trend of higher occurrence and death rates after decades of extensive research and therapeutic options. Traditional interventions are being used to increase survival, but each has its own limitations such as resistant tumors, how the body responds to treatment, and toxicity to other parts of the body.^1^ This has incited intensive interest in molecularly targeted therapies and immunotherapies to improve the treatment specificity while minimizing off-target toxicities. The MYC proto-oncogene is one of the most important oncogenic drivers that has been found to play a role in tumorigenesis.^2^ MYC controls diverse cellular events such as transcription, cell cycle, metabolism, and apoptosis. MYC dysregulation is linked to a wide range of hematological malignancies and solid tumors, and is a key focus in cancer biology and a highly promising drug target. Nevertheless, the nuclear localization, absence of enzyme activity and widespread interaction with numerous signalling pathways have long considered MYC to be undruggable and to pose unusual challenges to pharmacological intervention.

 Along with the developments in molecular oncology, the tumor microenvironment has emerged as one of the main determinants of the cancer progression and therapeutic response. Although T cell-based therapies have taken centre stage in the immuno-oncology scene, there is growing evidence of how B cells can play a tumor-promoting or tumor suppressing role.^3^ The contribution of B cells to antitumor immunity is via antigen presentation, antibody production, and antibody-dependent cellular cytotoxicity, but also includes regulatory subsets that inhibit immune activation processes via cytokine secretion and immunosuppressive signalling. Developments in single-cell sequencing technology have also revealed that there are different types of B cells in the tumor microenvironment and have led scientists to discover several new subsets of B cells that may have different prognoses and relevance for therapy.

 The biology of MYC and B-cell activity is of special interest because B-cells cannot develop and proliferate in the absence of MYC. Unregulated MYC activity is implicated in the pathogenesis of multiple B-cells associated malignancies such as Burkitt lymphoma and diffuse large B-cell lymphoma.^4^ Overlapping pathways provide a compelling rationale for developing strategies to exploit B-cell biology to target MYC or MYC-mediated biological activity as therapeutic candidates. Emerging therapeutic paradigms, including engineered B-cell platforms, intrabodies, nanobodies and antibody drug conjugates, continue to be positively examined for the purpose of overcoming the historic barriers of MYC targeting. Many recent reviews have comprehensively addressed MYC biology and MYC pharmacologic inhibition strategies; however, few consider unifying MYC signaling with the development of engineered and cloned B-cell immunotherapy platforms. Therefore, this review shifts from focusing on only inhibiting MYC to examining MYC-mediated immune reprogramming through modalities based on exploitation of MYC-driven tumor dependencies.

## Rationale for MYC as a Cancer Drug Target

 MYC is a key oncogenic driver for different cancers, but there’s more to its role in cancer than just controlling cell division (proliferation); MYC also influences the ability of cancers to present antigens and respond to the immune system (i.e., according to the “immunogenicity” of the tumor). MYC impacts antigen presentation and IFN signaling, as well as increases immune checkpoint protein, so MYC tumors effectively evade the immune response(s) including any potential for responding via the adaptive immune response (the humoral immune response). Therefore, when developing therapeutic approaches that target MYC, we need to consider MYCs role not only in inhibiting the growth of the tumor cells but also in restructuring the immune system, particularly regarding the humoral response.^5^ Multiple models of oncogenic propensity to the MYC protein show evidence that when MYC expression is genetically suppressed, tumors regress, which indicates that tumor cells are highly reliant on continued MYC activity. MYC not only stimulates cell division, but it also remodels cell metabolism to increase biosynthetic output, alters how DNA is replicated and repaired, and changes the expression of immune-related genes in order to promote immune evasion. Furthermore, these diverse functions of MYC suggest that inactivating MYC has the potential to able to adversely impact proliferation, metabolism, genomic integrity, and the immunogenicity of tumors. The long-standing challenges associated with developing agents that target MYC relate to its inherently disordered protein structure, and its many physiological functions raise significant concerns about the risk of normal tissue toxicity.^6^ The finding that cancers are generally more sensitive to changes in MYC level provides a basis to target MYC with therapeutic options, such as selective delivery, synthetic lethality, or transient inhibition. In addition to using classical active site engagement (i.e. through the direct targeting MYC), it has been appreciated that MYC can also be indirectly targeted by targeting transcriptional co-regulators or utilizing protein degradation modalities, which may reduce MYC function without requiring classical active site interaction. Collectively, these factors create a strong rationale for continued translational interest in therapies directed toward MYC, particularly in combination with immune directed therapies, as these therapies can capitalize upon the effects of MYC modifications on the antigens on tumor cells and on the tumor microenvironment. Functionally, MYC mediated changes in tumor antigenicity and immune signaling also represent a mechanistic basis through which immune therapeutic approaches targeting B-cell-mediated antibody production and antigen presentation may be effective against tumors driven by MYC.

## MYC in Cancer Biology

 MYC’s most important characteristic from a therapeutic perspective is its ability to regulate the immune system. When MYC is activated within a given tumor, it inhibits the ability to present antigens so that T-cells can’t recognize them and respond. MYC also has the ability to inhibit the signaling pathways activated by interferons, and it enhances the expression of ligands for immune checkpoints such as PD-1. Together these mechanisms promote immune escape from the immune system. The presence of MYC in tumors leads to the production of various cytokines and chemokines that promote the recruitment of immunosuppressive cell types into the microenvironment of tumors, contributing to the development of immune-resistant microenvironments. Therefore, the inhibition of MYC in tumors will likely reduce tumor growth as well as restore recognition by T-cells, highlighting the importance of combining MYC-targeted therapies with immunotherapies. MYC exerts wide impact on cancer hallmarks by restructuring transcriptional programs, metabolic flux, and interactions with the immune microenvironment. Comprehending these activities is essential to rational design of therapies that either directly impair MYC activity or exploit susceptibilities from MYC-dependent rewiring.

 MYC controls transcription through heterodimerization with MAX, binding to E-box motifs and recruiting transcriptional co-factors that control chromatin accessibility and elongation.^7^ Its activity is modulated at multiple layers including promoter occupancy, enhancer activation, and post transcriptional control. Epigenetic regulators such as bromodomain proteins, histone acetyltransferases and chromatin remodeling complexes act along with MYC to augment gene expression. Notably, the engagement of positive transcription elongation factors and cyclin-dependent kinases connects MYC functionality of transcriptional pause release and RNA polymerase processivity. Any alterations or changes in these auxiliary nodes indirectly suppresses MYC-directed transcription.

 The central theme of MYC biology is metabolic reprogramming. MYC activates glycolytic enzyme and glutaminolytic pathways, biosynthesis of nucleotides and lipid synthesis to fulfil the biosynthetic and energetic requirements of tumor cells that grow rapidly.^8^ This generates addictions to certain metabolic enzymes and carriers which can be used therapeutically. As an illustration, tumor cells with high MYC activity are often addicted to glutamine or hyper-reliant on nucleotide biosynthesis, create therapeutic entry points which can be leveraged through metabolic inhibitors or synthetic lethal strategies. MYC also programs immune modulation. It inhibits antigen presentation pathways, down regulates the responses of type I interferons and stimulates the secretion of cytokines and chemokines which favour an immunosuppressive microenvironment.^9^ In certain situations, the activity of MYC is associated with the expression of immune checkpoint ligands, and promotes the recruitment or development of suppressive myeloid cells and regulatory lymphocyte subsets. MYC inhibition, therefore can potentially not only slow proliferation, but also remodel tumor immune contexture such that it becomes vulnerable to immune therapies.

 Lastly, the role of MYC varies with hematologic malignancies and solid tumors. MYC has been commonly activated in B-cell-derived lymphomas by chromosome translocations or amplifications and directly mediates malignant change and progression. MYC activation of solid tumors is frequent, either by a signalling cascade or by a genomic mutation that raises the levels or stability of MYC. These differences have a therapeutic approach design implication since the cellular context determines both the downstream dependencies and the character of the immune interactions. These immune-modulatory functions of MYC extend beyond tumor-intrinsic growth control and directly influence the composition and functionality of tumor-infiltrating lymphocytes. This dual oncogenic and immunoregulatory role provides a rationale for integrating MYC targeting with B-cell-based immune engineering strategies.

## Tumor Microenvironment: B-Cells

 B-lymphocytes are increasingly recognized as important components of the tumor microenvironment that exert protumor and antitumor functions based on their spatial organization, phenotype and interaction with other immune components.^10^ Effector B cells are capable of presenting antigens to T cells and generating high-affinity antibodies which can mediate direct tumor clearance or recruit innate effector cells through antibody dependent cellular cytotoxicity and phagocytosis. Antibodies can also disrupt oncogenic signalling or deliver cytotoxic payloads when formatted as antibody-drug conjugates. In tertiary lymphoid structures, B cells support local immune responses and are associated in many studies with improved prognosis and responsiveness to immune checkpoint blockade.

 In order to promote MYC-induced immune modulation, B-cells within the tumor microenvironment have context-dependent roles to play. When functioning as effector B-cells, they provide antitumor immunity through antigen processing and antibody-mediated mechanisms. Conversely, regulatory B-cells promote immunosuppression using cytokine signaling mechanisms. In addition, MYC dysregulation impacts the signaling pathways of both tumor and tumor-infiltrating B-cells and the function of these cells, providing evidence for a bidirectional relationship between MYC, tumor-intrinsic signaling and the function of B-cells. This interplay between MYC, the function of B-cells and the tumor microenvironment suggests a potential mechanism for using B-cell based immune therapies to counteract MYC mediated immune evasion.

 Conversely, regulatory B cells produce immunosuppressive cytokines such as interleukin-10 (IL-10) and transforming growth factor beta (TGF-β) and can inhibit effector T-cell responses, promote regulatory T-cell expansion and modulate myeloid cell function to promote tumor progression.^11^ The duality of B-cell functions has been further clarified by single-cell profiling studies that reveal a spectrum of B-cell states within tumors, with distinct transcriptional programs and surface markers. These single-cell insights enable the identification of B-cell subsets associated with favorable or poor prognosis and drive strategies to either harness effector B-cells or deplete or reprogram regulatory B-cells. From a therapeutic standpoint, the prognostic and therapeutic relevance of B-cell infiltration depends on tumor type and context. In several carcinomas and melanoma, the presence of organized tertiary lymphoid structures and class-switched memory B-cells predicts better outcomes and greater benefit from immune checkpoint inhibitors indicating that B-cell targeted strategies can synergize with existing immunotherapies.^12^ In B-cell malignancies, the actual tumor cell is a B cell, thus B-cell malignancies require therapies to distinguish between the malignant B-cell populations from the prognostic, protective B-cell populations. Essentially, B-cells serve both as targets for and as tools of cancer therapy; therefore, an in-depth understanding of B-cell activity in specific tumor environments will lay the foundation for creating a rationally designed MYC-targeted immunotherapy. Since MYC regulates B-cell proliferation, germinal center dynamics, and B cell-related immune signaling, it is plausible that tumor-associated dysregulation of MYC will directly affect B-cell phenotype in the tumor microenvironment. These observations indicate a complex relationship between MYC and B-cell activities; further, genetically modified B-cells may be therapeutically deployed against MYC mediated oncogenic regulatory programs.

## MYC and B-Cell Development

 MYC serves as a key regulator of normal B-cell development, where it regulates progenitor proliferation, somatic hypermutation, germinal centre dynamics, and differentiation.^13^ MYC expression is highly regulated throughout B-cell development and occurs in dynamic pulses which support proliferation and selection of B-cells in germinal centres, followed by subsequent downregulation during the phase of differentiation into long-lived plasma cells or memory B cells. MYC levels corroborate with the regulatory mechanisms controlling proliferation, DNA repair, and apoptotic sensitivity and protect against malignant transformation of B-cells. Loss of MYC control by chromosomal translocations, gene amplification, or aberrant upstream signalling may lead to unchecked proliferation and survival of B-cells which is the basis of many B-cell lymphomas. Classic examples are Burkitt lymphoma, in which t (8;14) translocations position MYC next to immunoglobulin enhancers, that promote constitutive expression of MYC.^14^ MYC overexpression is frequently accompanied by changes in other oncogenic pathways such as activation of PI3K, AKT, JAK STAT or NF-κB signalling pathways that together promote malignant phenotypes and affect therapeutic responses in diffuse large B-cell lymphomas and other malignancies.

 Animal models have proven very valuable in assessment of the role of MYC dysregulation in B-cell malignancies, and analysing the possible interventions.^15^ Conditional MYC transgenic models closely recapitulate disease phenotypes and demonstrate that modulation of MYC activity can trigger tumor regression through suppressive mechanisms. However, these responses are often transient, as tumors may recur due to the activation of compensatory pathways. Additionally, using these models, understanding of contextual-dependent vulnerabilities related to interaction between MYC and PI3K, AKT & NF-κB pathways as well as the availability of therapeutic strategies open up avenues for the use of combination strategies. Therefore, it is essential that patients be stratified on the basis of co-occurring pathway varies and immune microenvironment to enhance therapeutic benefit and minimize toxicities.

###  MYC as an Immune-Orchestrating Oncogene: A Framework for B-Cell Engineering

 The accumulation of knowledge indicates that MYC functions as a critical orchestrator of tumor-immune interactions and is the classical master regulator of proliferation/metabolism. MYC activity has been found to inhibit antigen presentation by repressing elements of the major histocompatibility complex (MHC) and impairing the function of signalling cascades downstream of interferon receptors. As antigen presentation capacity is decreased, it reduces visibility of tumors to cytotoxic lymphocytes, which in turn contributes to immune escape. Transcriptional repression of genes involved with antigen processing and attenuation of type I IFN responses in MYC-transformed malignancies create a privileged immune compartment conducive for tumor survival.

 Besides affecting antigen presentation, MYC also regulates immune checkpoints and contributes to the overall immunosuppressive microenvironment within tumors. MYC activity elevates expression of immune checkpoint ligands, including PD-L1, in select tumors leading to T-cell exhaustion and decreased antitumor immunity. MYC-driven tumors also produce cytokine and chemokine profiles that alter the recruitment of suppressive myeloid cell populations and regulatory T cells to the tumor site. These cytokine changes create an immunosuppressive tumor microenvironment by preventing effective adaptive immune responses and fostering pro-tumoral inflammation. Thus, MYC is not only an oncogenic driver within transformed cells; it regulates the immune ecosystem around the tumor.

 Importantly, as discussed, MYC also plays an essential physiological role in B-cell development, germinal centre dynamics, and clonal expansion. Dysregulated MYC signalling affects not only tumor cells but also the functionality of B cells within the tumor microenvironment. This intersection suggests that MYC status may serve as a biological determinant for immunotherapy design. In MYC-driven tumors characterized by reduced antigen presentation and immunosuppressive cytokine signalling, engineered B-cell platforms could be strategically deployed to restore humoral immunity, provide sustained antibody delivery, enhance antigen presentation, or modulate local immune networks. In this context, MYC expression patterns may guide the selection and optimization of B-cell–based therapeutic interventions.

 MYC should be considered not solely as a molecular drug target but as a biological determinant guiding the design of humoral immunotherapy platforms. By reframing MYC as an immune-orchestrating oncogene rather than exclusively a transcriptional amplifier, this conceptual approach integrates oncogenic signalling with engineered B-cell strategies. This repositioning distinguishes the present review from prior MYC-focused summaries and provides a translational framework for MYC-guided immune reprogramming in cancer therapy.

###  Conceptual Framework for MYC-Guided Immune Reprogramming

 Recent advances in both clinical and research settings suggest myc should be considered differently than just an immune regulator. MYC should be classified as a more formalized translational framework, to better define tumors based on functionality (tumor type) and their immune phenotype (how they respond to the immune system); therefore, the goal would be to create a unified set of classifications so that researchers can develop better therapeutic strategies for MYC-driven tumors. A simplified set of hypothetical classifications can be developed.^16,17^

 Tumors that are MYC-high (known as cold phenotype) exhibit low MHC expression, low levels of type 1 IFN signalling, high levels of immune checkpoints (eg PD-L1) and an abundance of immune suppressive cell types. Because of their inability to process immune responses, MYC-high tumors will generally have poor responses to typical immune therapies.^18,19^ Tumors of the MYC-intermediate type (modified phenotype) have a moderate amount of immune suppression and both antigen processing and immune cell infiltrate levels can vary. Treatment for MYC-intermediate tumors may use therapies such as immunotherapy in combination with therapies that target MYC signaling. Tumors of the MYC-low type (active phenotype) are those that have intact antigen processing, are associated with a large number of immune cells, and have a maximum response rate to immunotherapy due to their high baseline levels.^19^

 Using this system would allow you to correlate MYC status, immune phenotype, and therapeutic approach in the following way:

 MYC-high → Primarily associated with immune escape → Treat with CAR-B cells and intrabody therapies, as well as immunotherapy with refractory immune checkpoint inhibitors;

 MYC-intermediate → Mixed phenotype → Use BET/CDK inhibitors in conjunction with immunotherapy;

 MYC-low → Immune response → Use traditional immunotherapy methods, with the potential for MYC targeting.

 The creation of an MYC biological framework supports MYC’s role as not only a potential target for therapy but also as a biological determinant - providing a strong argument for the use of engineered B-cell platforms to be used in MYC-high tumours, to support restoration of humoral immunity, to increase tumour exposure to antigen processing, and to provide a method to target delivery of products via antibody-mediated approaches. A structured approach to MYC biology provides a translational platform that links MYC biology to precision immunotherapy and allows improved classification of patients and more rational designs for combination therapies. The MYC-guided immune reprogramming framework illustrating the relationship between MYC expression levels, immune phenotype, and therapeutic strategies as shown in Figure 1.

**Figure 1:**
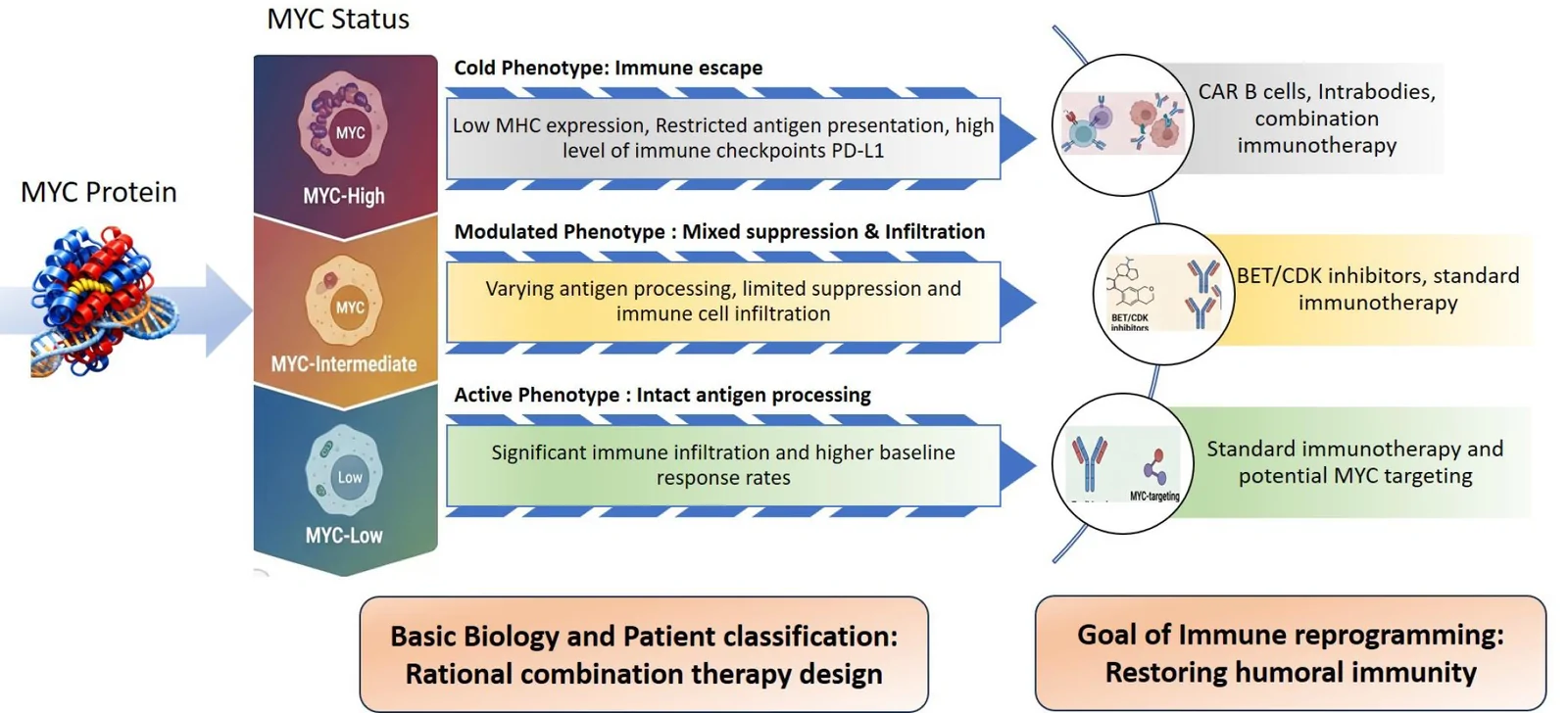
MYC-guided immune reprogramming framework illustrating the relationship between MYC expression levels, immune phenotype, and therapeutic strategies

## Therapeutic Approaches to MYC

###  Indirect Transcriptional Suppression (BET and CDK Inhibitors)

 Indirect MYC targeting through transcriptional regulators remains the most clinically advanced strategy. BET inhibitors such as OTX015 (MK-8628; NCT01713582, NCT02259114) and CPI-0610 (NCT02158858) suppress MYC transcription by disrupting BRD4-mediated super-enhancer function. Early-phase trials in hematologic malignancies, including diffuse large B-cell lymphoma (DLBCL) and acute myeloid leukemia, demonstrated partial responses in molecularly selected subsets; however, dose-limiting toxicities including thrombocytopenia, gastrointestinal toxicity, and fatigue—have constrained durable dosing.

 Similarly, CDK9 inhibitors (e.g., alvocidib; NCT03298984) and CDK7 inhibitors reduce transcriptional elongation and destabilize MYC-driven transcriptional programs. Although, biological activity has been observed with them, responses to these agents remain heterogeneous and frequent resistance development is seen mediated by compensatory signalling pathways. These limitations emphasize that indirect transcriptional suppression as a single approach may not be suffice and is likely to require rational combination strategies, particularly with immune checkpoint inhibitors or DNA damage response (DDR) inhibitors. Overall, among currently tested approaches, indirect transcriptional suppression is the most clinically validated but limitations of toxicity and modest single-agent efficacy are observed.^20^

###  Direct MYC–MAX Disruption

 Direct targeting of MYC via disruption of MYC–MAX heterodimerization appears promising because it addresses the core oncogenic function of MYC. Small molecules such as 10058-F4 have demonstrated proof-of-concept in preclinical models; however, poor potency, instability, and pharmacokinetic limitations were observed and has hampered their clinical translation. Fragment-based drug discovery and structural refinement efforts are ongoing to produce clinically viable MYC–MAX disruptor but none of the molecules has entered advanced trials yet. Although, this approach is mechanistically sound, it is still in preclinical phase and is technically challenging.^21^

###  RNA-Based MYC Suppression

 RNA interference strategies, including antisense oligonucleotides (ASOs) and siRNA-based constructs can lead to sequence-specific MYC suppression. Preclinical xenograft models have demonstrated tumor growth reduction with RNA interference strategies but delivery, stability, and innate immune activation of these biologics are usually the major barriers to translation. Recent lipid nanoparticle and mRNA platform advancements have improved feasibility, but clinical validation of this approach in MYC-driven cancers is still scanty. At present, RNA-based MYC targeting appears promising but at an early stage relative to transcriptional inhibitors.^22^

###  Synthetic Lethality and Metabolic Targeting

 MYC-driven tumors exhibit replication stress and metabolic dependencies, particularly on ATR/CHK1 signaling and glutamine metabolism. ATR inhibitors (e.g., ceralasertib; NCT03770429) are being evaluated in combination settings. Synthetic lethal approaches may offer superior tumor selectivity compared to direct MYC inhibition, especially in genomically unstable malignancies. However, biomarker-driven patient selection is essential, and predictive markers of MYC dependency remain underdeveloped. Among emerging strategies, synthetic lethality approaches appear particularly promising due to tumor-selective vulnerability exploitation, though clinical validation is ongoing.^23^

###  Targeted Protein Degradation (PROTACs)

 PROTAC-based degradation of MYC regulators (e.g., BET degraders such as ARV-825) may overcome resistance observed with classical inhibitors by inducing sustained protein depletion. These modalities demonstrate enhanced potency in preclinical models but are in early translational stages.^24,25^ Collectively, these approaches represent a paradigm shift in MYC drug discovery, as the perception of MYC as an inaccessible target has been replaced by a realistic one as an actionable treatment target. Table 1 discusses the various therapeutic strategies targeting MYC.

**Table 1:** Therapeutic Strategies Targeting MYC: Mechanisms, Advantages and Limitations

**Approach**	**Mechanism**	**Advantages**	**Limitations**	**Examples Agents**	**Reference**
Transcriptional suppression	Inhibit bromodomain proteins or transcriptional CDKs to reduce MYC expression	Indirect suppression of MYC and downstream programs	Global transcriptional effects potential toxicity resistance	BET inhibitors CDK9 inhibitors	^26^
MYC MAX disruption	Prevent heterodimer formation and DNA binding	Directly targets core MYC function	Intracellular delivery potency challenges	Small molecules stapled peptides	^27^
RNA based	siRNA antisense oligonucleotides miRNA mimics reduce MYC mRNA	High specificity potential for sequence guided targeting	Delivery stability innate immune activation	siMYC ASO constructs	^28^
Synthetic lethality	Target DDR or metabolic dependencies induced by MYC	Tumor selective killing exploits collateral vulnerabilities	Patient heterogeneity need for biomarkers	ATR CHK1 inhibitors metabolic inhibitors	^29^
PROTACs and degraders	Recruit E3 ligases to degrade MYC or stabilizing cofactors	Potent targeted protein removal novel modality	Early-stage delivery selectivity concerns	PROTAC molecules targeting regulators	^30^
Intrabodies nanobodies	Intracellular antibodies bind sequester or degrade MYC	High specificity adaptable to degradation tags	Intracellular delivery and stability	Viral vector delivered intrabodies	^31^

 These limitations highlight the need for complementary immune-based strategies, particularly those capable of sustained biologic delivery within the tumor microenvironment. Many MYC-directed therapeutic modalities are in development or have demonstrated impressive in-vitro performance but have not produced consistent clinical success thus far, demonstrating the need for rational combinations and improved patient selection based on biomarkers.

## Antibody and B-Cell-Based MYC Targeting

###  Intrabodies and Nanobodies

 Intracellular antibodies targeting MYC offers a novel strategy to overcome the “undruggable” paradigm. Nanobody platforms have structural advantages like small size and enhanced intracellular stability which can help them exert their effects at desired location. Preclinical lymphoma models have demonstrated proof-of-concept of MYC suppression and antitumor effects of this modality. However, delivery efficiency, intracellular persistence, and safety concerns for them persists. None of the clinical trials have yet validated the effectiveness and safety of this strategy. Thus, intrabody-based MYC targeting strategy is innovative but still in its infancy.^32^

###  Bispecific Antibodies and Peptide–MHC Targeting

 Targeting MYC-derived peptide–MHC complexes via TCR-mimetic or bispecific antibodies is a rational approach to convert intracellular oncogenic signalling into immune-recognizable targets. However, there are several challenges to this approach which limit its therapeutic success, such as peptide presentation heterogeneity, HLA restriction and risk of antigen escape. Although this approach is expected to enhance tumor specificity, it will require robust antigen validation and discriminatory patient stratification.^33^

###  Antibody–Drug Conjugates (ADCs)

 MYC is located intracellularly and ADC modalities exploit the surrogate surface markers upregulated in MYC-driven tumors. This indirect strategy may be crucial in providing selective cytotoxicity. However, identification of robust MYC-correlated surface antigens is challenging and off-target toxicities continue to pose a concern. ADCs are clinically validated in oncology broadly but they have not yet been specifically optimized for MYC-driven malignancies.^34^

###  Engineered B-Cells and CAR-B Platforms

 Engineered B-cell platforms are a conceptual expansion beyond CAR-T therapies. CAR-B cells allow for antibody secretion, antigen presentation and immune modulation which is different from CAR-T cells. The potential beneficence over CAR-T therapies is based on a possibly reduced risk of cytokine release syndrome, providing long-term humoral immunity and physiological homing to lymphoid tissues. However, CAR-T therapy has received multiple FDA approvals and CAR-B therapy is still in early preclinical stages. Manufacturing standardization, safety switches, and transformation risk must be addressed before clinical translation.^35^ Table 2 details a comparative perspective between the different strategies.

**Table 2:** Comparative summary of MYC-targeting therapeutic strategies

**Strategy**	**Clinical Maturity**	**Strength**	**Limitation**
BET/CDK inhibitors	Early clinical	Feasible, validated	Toxicity, resistance
Synthetic lethality	Early clinical	Tumor selectivity	Biomarker need
CAR-T	Clinically approved	Potent cytotoxicity	CRS, exhaustion
CAR-B	Preclinical	Immune orchestration	Early-stage, unproven
Intrabodies/Nanobodies	Preclinical	Intracellular MYC binding	Delivery Challenges
PROTACs	Preclinical	Protein degradation	Early-stage development

 Thus, B-cell–based approaches are mechanistically compelling but currently less clinically mature than small-molecule or CAR-T approaches.^36^ Cloned B-cell populations present a unique therapeutic architecture different from T-cell cytotoxic platforms. CAR-T cells primarily mediate direct tumor cell killing while engineered B cells can function as continuous antibody-producing bioreactors. Further B-cells are simultaneously participating in antigen presentation, and microenvironment modulation. This multifunctional capacity may be particularly advantageous in MYC-driven tumors characterized by immune suppression and antigen heterogeneity.

###  Comparative Analysis of MYC-Targeting Therapeutic Modalities

 There are significant differences between groups of therapies that are actually MYC related in terms of how they work, how they were developed in the clinic, as well as how they may possibly be used as personalized medicine for patients. Currently available therapeutics include small molecules like bromodomain and E3 ligase inhibitors, cyclin-dependent kinase (CDK) inhibitors and mRNA inhibitors with regards to MYC. All of these therapies have been somewhat successful in clinics but are limited to treatment only by their systemic toxicity to non-targeted cells, as well as their poor selectivity to specifically targeted cells versus developing resistance via compensatory pathways that develop with time. In contrast, there is another strategy that is emerging that takes advantage of the MYC-related characteristics of derived tumors called “synthetic lethality.” Synthetic lethality utilizes the MYC-driven vulnerabilities of tumors to enable the safe elimination of tumour cells while sparing healthy ones. However, the success of synthetic lethality depends critically on the availability and utility of optimal biomarkers for stratifying patients.^30^

 Mechanistically, immune-based therapies have a distinct mechanism of action. CAR-T cell therapies have demonstrated efficacy against malignant cell killing, as well as treatment of hematologic malignancies; however, the use of CAR-T is limited due to the adverse effects of cytokine release syndrome, T cell fatigue, and neurotoxicity. In comparison to CAR-T, engineered B-cell (CAR-B) therapies provide several relative advantages; including maintaining the ability to produce antibodies for longer periods of time, presenting antigens to T cells, and modifying the tumor microenvironment. These features could potentially be advantageous in treating MYC-driven solid tumors with antigen heterogeneity and immune suppression. However, CAR-B is currently in the developmental phase using preclinical models and requires additional research and development for areas related to product manufacturing, safety testing, and long-term stability of CAR-B products.

 Intrabodies and nanobodies are types of intracellularly target therapies that provide extraordinary specificity for MYC; however, they have significant limitations as there are no proven clinical outcomes, nor can therapeutically beneficial forms be delivered to patients. Small molecule inhibitors are less complex to manufacture and deliver than biological therapies; however small molecules do not provide the opportunity for prolonged, therapeutic modulation of the immune system.^28^

 Therapeutic approaches to cancer should be contextualized by the environment of the cancer cells. CAR-T therapy is the most established method for the treatment of MYC-driven hematologic malignancies; it has demonstrated the greatest clinical utility and the most evidence for use. In contrast, therapies that seek to inhibit MYC activity and utilize other immune-based treatment options such as CARB or antibody therapies against solid tumours with an immune-cold phenotype will likely yield the best results when used in combination with one another. Therefore, it is more reasonable to consider these different therapeutic modalities as complementary rather than mutually exclusive methods by which to develop a rational basis for the development of combination therapies.

## Preclinical and Clinical Developments

 Clinical translation of MYC targeting has largely centred on indirect transcriptional inhibitors. BET inhibitors such as OTX015 (NCT01713582) and CPI-0610 (NCT02158858) demonstrated modest response rates in hematologic malignancies, but thrombocytopenia and gastrointestinal toxicity limited dosing. CDK inhibitors (e.g., alvocidib; NCT03298984) showed apoptosis induction in MYC-driven leukaemia’s but required combination regimens for durable responses. ATR inhibitors (e.g., ceralasertib; NCT03770429) are being evaluated in biomarker-selected settings exploiting replication stress in MYC-high tumors. The overall clinical landscape suggests that MYC dependency is heterogeneous and single-agent MYC modulation may yield partial responses; hence, combination regimens are likely required for efficient therapeutic intervention.

 Antibody and B-cell-based MYC strategies remain largely preclinical. Early xenograft studies demonstrate feasibility, but human data are lacking. Therefore, translational enthusiasm should be balanced with recognition of developmental stage.^27,37^ Clinical advancement in the field has been mainly in testing indirect MYC inhibitors like BET and CDK inhibitors. There are multiple early-phase trials for these inhibitors on patients having a MYC-driven malignancies. Findings so far have shown some biological activity in selected situations, and have also shown difficulties of toxicity and heterogeneous responses that point to the importance of reliable biomarkers. Combination of transcriptional inhibitors with targeted drugs, DNA damage response drugs or immune therapy trials are in progress and are a logical direction forward.^38^ Antibody and B-cell-based interventions are at an earlier, largely preclinical phase of clinical development as intracellular antibody delivery platforms and engineered cell therapies are expected to be utilized in the near future as delivery technologies make progress. To precisely monitor the progress of clinical trials, it is necessary to have up to date registries and trial identifiers that should be reported in manuscripts to provide transparency and reproducibility. Table 3 demonstrates preclinical and clinical advances in MYC targeting.

**Table 3:** Preclinical and Clinical Advances in MYC Targeting

**Strategy**	**Model Trial**	**Key Findings**	**Clinical Status**	**Reference**
BET inhibition	Preclinical lymphoma models	Tumor regression variable depending on context	Early-phase clinical trials ongoing	^39^
CDK inhibition	Hematologic and solid tumor models	Induction of apoptosis transcriptional suppression	Phase I II trials with toxicity monitoring	^40^
RNA based MYC knockdown	In vitro and xenograft models	Reduced tumor growth proof of concept	Preclinical development	^41^
Intrabody delivery	Mouse B-cell lymphoma models	MYC inhibition antitumor activity	Preclinical	^42^
CAR B and engineered B-cells	Xenograft models	Humoral responses and tumor control	Early stage preclinical	^43^

 Translational gaps still exist, such as strong biomarkers of MYC dependence that have standardized procedures of measuring MYC activity, and improved methods of delivering intracellular biologics and nucleic acids are needed. There is a need to fill these gaps in order to transform promising preclinical candidates into sustained clinical benefit as shown in Table 4.

**Table 4:** Translational Mapping of MYC Status to Therapeutic Strategies

**Tumor Type**	**MYC Alteration**	**Immune Phenotype**	**Recommended Strategy**
Burkitt lymphoma	MYC translocation	Immune-variable	CAR-T, emerging CAR-B, intrabodies
DLBCL	MYC amplification/overexpression	Immune-suppressed	BET/CDK inhibitors + immunotherapy
Acute leukemias	MYC dysregulation	Immune heterogeneous	CDK inhibitors + combination therapy
Solid tumors (e.g., breast, lung)	MYC overexpression	Immune-cold	MYC inhibitors + checkpoint blockade
MYC-high tumors (pan-cancer)	Functional MYC activation	Low antigen presentation	CAR-B, antibody-based approaches

## Challenges and Limitations

 There are basic and practical problems in the design and implementation of MYC-directed therapies. Heterogeneity in tumors across patients and within tumor results in varying degrees of dependency upon MYC and there is a possibility of selection of the resistant clones that do not depend on MYC.^44^ Immunotherapeutic approaches enhancing recognition of peptide-MHC complexes or MYC associated surface markers in MYC-driven malignancies may be compromised by antigen escape. The most critical aspects of RNA-based therapeutics, intrabodies and engineered B-cell products are delivery and stability as it is challenging to achieve measurable concentrations in tissues and cells of interest without having issues of immune recognition of delivery vehicles. Off-target toxicity and autoimmunity are safety issues of importance since MYC plays crucial roles in normal regenerative tissues and immune based therapies that induce widespread activation of humoral or cellular responses and pose the risk of damaging normal tissues bearing common antigens. Measures to reduce toxicity concerns with MYC-directed strategies include tumor targeted delivery, conditional expression systems, transient dosing schedules and safety switches in engineered cells.

 The other significant shortcoming is that there are currently very few validated biomarkers to determine patients who are most likely to respond to MYC-targeted strategies.^45^ MYC gene amplifications or translocations are beneficial but they do not comprehensively and quantitatively capture functional MYC activity that is affected by post translational control and parallel pathway changes. Patient stratification based on comprehensive biomarker information should be used to enhance response rates and limit exposure of unlikely responders to possible toxicities.

 Robust biomarker-driven patient stratification is required to successfully translate clinical MYC-targeted therapies into practice. Traditional markers, such as MYC amplification or overexpression of MYC products, do not have sufficient predictive value for MYC activity in the clinical setting. As such, there is a pendulum swing in terms of understanding the activity of MYC using transcriptional profiling (that is, MYC-dependent transcriptional signatures), epigenetic profiling, and pathway activation states. There is also a growing interest in understanding the role of immune-related biomarkers (e.g., PD-L1 expression, MHC expression levels, and pattern of immune cell infiltration) as complementary markers of MYC activity to assist in guiding treatment choices for patients with MYC-dependent tumors. Ultimately, the integration or correlation of molecular and immune-based biomarkers will be essential to optimally identify patients with MYC-dependent tumors who are most likely to benefit from MYC-targeted and MYC-guided immunotherapeutic strategies.

###  Why MYC-Targeting Strategies Face Clinical Limitations

 Many advances have been made towards MYC-targeting therapeutic strategies, but they are limited in their ability to cross over into the clinical setting because of many biological and technological barriers. MYC-targeted cancer treatment faces a multitude of challenges that limit the ability of MYC-targeted therapies to work effectively in the clinic, with one of the largest challenges lying in the intrinsic heterogeneity of MYC dependency among individual tumors. Although some tumors highly express MYC, the majority of these tumors do not reveal equal dependence upon MYC signalling for proliferation, and thus, some tumors are able to quickly adapt to MYC inhibition through the activation of compensatory oncogenic signaling pathways within the tumor. This heterogeneity/compensatory ability of many tumors limits the efficacy of MYC-targeted therapies in the clinical setting.

 Resistance mechanisms further complicate the effective treatment of MYC-dependent tumors. Many of the MYC inhibitors that are currently being developed are either directly inhibiting MYC or one of its co-signaling pathways. The inhibition of MYC and/or associated co-signaling pathways will frequently activate alternative survival signaling pathways, including those involving the PI3K/AKT pathway, NF-κB pathway, and MAPK pathway. This rewiring allows tumor cells that have been inhibited by MYC to go around MYC inhibition by using alternative survival pathways, therefore allowing for continued proliferation. Additionally, because of the high levels of genomic instability found in MYC-driven tumors, there is ongoing clonal evolution and selection of tumor populations resistant to MYC inhibition throughout treatment.

 From an immunological perspective, MYC-associated immune evasion adds another layer of complexity. MYC impacts antigen presentation and interferon signaling, leading to a decrease in the efficacy of immunotherapy, especially in immune-cold tumors. The loss of antigen presentation and heterogeneity within the peptide-MHC complexes can also diminish the effectiveness of targeted immunotherapies such as bispecific antibodies or CAR-based strategies.

 There are still large technological barriers that limit the successful implementation of MYC-targeted therapy options. The low efficiency of intracellular delivery of RNA-based therapeutics, antibodies, and nanobodies limits their use in MYC-targeted therapies and promote off-target immune reactions. Further, engineered B-cell therapies must overcome challenges associated with the complexity of manufacturing, safety concerns, and long-term stability of these cellular therapy products. The CAR-based T-cell therapy products with established clinical protocols prove to be much simpler than currently engineered B-cell therapy products, which currently lack standardized manufacturing and regulatory processes.

 Lastly, a major translational barrier to the successful clinical use of MYC-targeted therapies is the absence of standardized and robust biomarkers for MYC activity. Current methodologies for measuring MYC activity relies predominantly on measuring MYC gene amplification or MYC protein expression; neither method accurately defines functional MYC activity for appropriate patient stratification. Without sufficient specificity for selecting patients who will respond to MYC-targeting therapies, variability and unpredictability in clinical responses to MYC-targeting therapies will persist.

 The challenges associated with MYC-targeting therapies illustrate that MYC-targeting therapies remain conceptually ideal, but successful implementation in the clinical setting, will require integrated approaches that will include all three elements of molecular targeting, immune modulation, and using biomarkers for patient stratification.

## Future Directions and Perspectives

 The integration of MYC-targeting agents with immune checkpoint inhibitors, DNA damage response (DDR) inhibitors, and metabolic therapies represents a rational and promising therapeutic direction. The successful translation in clinics will depend on robust biomarkers that reflect MYC transcriptional activity rather than relying on gene amplification status. Molecularly stratified and adaptive trial designs, incorporating integrated immune monitoring and standardized MYC activity assays, will be essential to optimize combination strategies and dosing paradigms.^46^

 Engineered B cell therapies and intrabody platforms remain promising but are still in early experimental stages. Advances in synthetic biology and CRISPR-based engineering are enabling increasingly precise reprogramming of B-cells to function as antibody-producing cells, antigen-presenting cells, or modulators of the tumor microenvironment, while incorporating safety safeguards to mitigate the risk of malignant transformation. Concurrently, technological progress in intracellular delivery systems—particularly lipid nanoparticles, mRNA platforms, and targeted viral vectors—has demonstrated the feasibility of in vivo nucleic acid and biologic delivery, with further optimization expected to expand applications to intrabody and nanobody-based approaches.^47^

 MYC-driven tumors may need to be treated with multiple drug approaches if long-term clinical improvements are sought. As MYC is frequently modulated by pathway changes, the single agent inhibition of MYC by existing drugs will not produce effective results because of resistance mechanisms activated through pathway alterations. Therefore, rational combinations of drugs are under investigation. For example, these combinations may include the use of immune checkpoint inhibitors, DNA damage response pathway inhibitors and metabolic modulators.

 Combination therapy utilizing the combination of BET inhibitors and checkpoint inhibitors has the potential to enhance the immuno-physical properties of tumors. Furthermore, combinations of CAR and B-cell therapies may provide continuous immune pressure on the tumor. Therefore, when creating combination therapy regimens, it is important to take into consideration (1) MYC status; (2) tumor type; and (3) the patient’s immune system in relation to the tumor immune response in order to develop the most effective combination therapy regimen and the least toxicity.

 Collectively, these innovations, coupled with the development of reliable functional biomarkers of MYC activity, will accelerate rational patient selection and trial design. Nonetheless, broad clinical implementation remains premature, and cautious optimism is warranted as translational validation continues.

## Conclusion

 The review underscores the conjunction between MYC-driven oncogenic signaling and engineered B-cell immunotherapy by highlighting MYC status as a guiding parameter for immune reprogramming rather than solely as a transcriptional target. It aims to kindle the emergence of translational strategies integrating humoral engineering with oncogene-directed therapy. MYC continues to be a vital yet therapeutically challenging oncogenic driver. Indirect transcriptional inhibitors are currently the most clinically advanced MYC-modulating strategies, though limited by toxicity and resistance. Synthetic lethality approaches and targeted protein degradation technologies are potential avenues but need a solid biomarker-guided validation. Antibody- and B-cell–based MYC targeting platforms broaden the immunotherapeutic options but still remain largely at preclinical stage. Although these developments are conceptually transformative, their clinical translation will mandate addressing the delivery, safety, and scalability challenges. Strategic combination regimens, discriminatory patient stratification, and advances in intracellular biopharmaceuticals delivery will determine whether MYC-directed immunotherapeutic approaches achieve long lasting clinical impact. Future clinical success will depend on integrating MYC-targeted therapies with biomarker-driven patient selection and rational combination immunotherapy strategies.

## Competing Interests

 No Competing Interests to Declare.

## Ethical Approval

 Not Applicable.

## References

[1] Zafar A, Khatoon S, Khan MJ, Abu J, Naeem A (2025). Advancements and limitations in traditional anti-cancer therapies: a comprehensive review of surgery, chemotherapy, radiation therapy, and hormonal therapy. Discov Oncol.

[2] Kontomanolis EN, Koutras A, Syllaios A, Schizas D, Mastoraki A, Garmpis N (2020). Role of oncogenes and tumor-suppressor genes in carcinogenesis: a review. Anticancer Res.

[3] Qin Y, Lu F, Lyu K, Chang AE, Li Q (2022). Emerging concepts regarding pro- and anti-tumor properties of B cells in tumor immunity. Front Immunol.

[4] Ott G, Rosenwald A, Campo E (2013). Understanding MYC-driven aggressive B-cell lymphomas: pathogenesis and classification. Blood.

[5] Wang C, Zhang J, Yin J, Gan Y, Xu S, Gu Y (2021). Alternative approaches to target MYC for cancer treatment. Signal Transduct Target Ther.

[6] Kumar D, Sharma N, Giri R (2017). Therapeutic interventions of cancers using intrinsically disordered proteins as drug targets: c-MYC as model system. Cancer Inform.

[7] Jakobsen ST, Siersbæk R (2025). Transcriptional regulation by MYC: an emerging new model. Oncogene.

[8] Goetzman ES, Prochownik EV (2018). The role for Myc in coordinating glycolysis, oxidative phosphorylation, glutaminolysis, and fatty acid metabolism in normal and neoplastic tissues. Front Endocrinol (Lausanne).

[9] Yang L, Li A, Lei Q, Zhang Y (2019). Tumor-intrinsic signaling pathways: key roles in the regulation of the immunosuppressive tumor microenvironment. J Hematol Oncol.

[10] Downs-Canner SM, Meier J, Vincent BG, Serody JS (2022). B cell function in the tumor microenvironment. Annu Rev Immunol.

[11] Schwartz M, Zhang Y, Rosenblatt JD (2016). B cell regulation of the anti-tumor response and role in carcinogenesis. J Immunother Cancer.

[12] Fridman WH, Meylan M, Petitprez F, Sun CM, Italiano A, Sautès-Fridman C (2022). B cells and tertiary lymphoid structures as determinants of tumour immune contexture and clinical outcome. Nat Rev Clin Oncol.

[13] De Silva NS, Klein U (2015). Dynamics of B cells in germinal centres. Nat Rev Immunol.

[14] Woroniecka R, Rymkiewicz G, Szafron LM, Blachnio K, Szafron LA, Bystydzienski Z (2022). Cryptic MYC insertions in Burkitt lymphoma: new data and a review of the literature. PLoS One.

[15] de Jonge AV, Mutis T, Roemer MG, Scheijen B, Chamuleau ME (2020). Impact of MYC on anti-tumor immune responses in aggressive B cell non-Hodgkin lymphomas: consequences for cancer immunotherapy. Cancers (Basel).

[16] Casey SC, Baylot V, Felsher DW (2017). MYC: master regulator of immune privilege. Trends Immunol.

[17] Nirala BK, Yustein JT (2025). The role of MYC in tumor immune microenvironment regulation: insights and future directions. Front Biosci (Landmark Ed).

[18] González-Larreategui Í, Valdés-Bango Martín M, Casacuberta-Serra S, Soucek L (2026). MYC at the tumor-immune interface: mechanisms of immune escape and immunotherapy resistance. Front Immunol.

[19] Wu B, Zhang B, Li B, Wu H, Jiang M (2024). Cold and hot tumors: from molecular mechanisms to targeted therapy. Signal Transduct Target Ther.

[20] Lüscher B, Vervoorts J (2012). Regulation of gene transcription by the oncoprotein MYC. Gene.

[21] Chaidos A, Caputo V, Karadimitris A (2015). Inhibition of bromodomain and extra-terminal proteins (BET) as a potential therapeutic approach in haematological malignancies: emerging preclinical and clinical evidence. Ther Adv Hematol.

[22] Kiessling A, Sperl B, Hollis A, Eick D, Berg T (2006). Selective inhibition of c-MYC/Max dimerization and DNA binding by small molecules. Chem Biol.

[23] Bajan S, Hutvagner G (2020). RNA-based therapeutics: from antisense oligonucleotides to miRNAs. Cells.

[24] Gajek A, Gralewska P, Marczak A, Rogalska A (2021). Current implications of microRNAs in genome stability and stress responses of ovarian cancer. Cancers (Basel).

[25] Pan M, Fu Z, Hou H, Yang C, Li J (2025). Proteolysis-targeting chimera (PROTAC): a revolutionary tool for chemical biology research. Small Methods.

[26] van der Noord VE, van de Water B, Le Dévédec SE. Targeting the heterogeneous genomic landscape in triple-negative breast cancer through inhibitors of the transcriptional machinery. Cancers (Basel) 2022;14(18). doi: 10.3390/cancers14184353. PMC9496798 36139513

[27] Weber LI, Hartl M (2023). Strategies to target the cancer driver MYC in tumor cells. Front Oncol.

[28] Chakkyarath V. Applications of advanced ASOs, RNA mimics, and others in RNA-based therapeutics. In: Sharma NK, Chand Dakal T, Maurya PK, Wadhwa G, eds. RNA-Based Cancer Therapeutics: Techniques, Applications and Challenges in Prognosis. Singapore: Springer; 2025. p. 35-62. doi: 10.1007/978-981-96-3456-9_3.

[29] Fernandez A, Artola M, Leon S, Otegui N, Jimeno A, Serrano D (2025). Cancer vulnerabilities through targeting the ATR/Chk1 and ATM/Chk2 axes in the context of DNA damage. Cells.

[30] Dale BL. Advancing Targeted Protein Degradation: Mechanistic Insight and Characterization of EZH2 Degradation as Triple Negative Breast Cancer Therapeutic [dissertation]. Icahn School of Medicine at Mount Sinai; 2024.

[31] Zhang C, Ötjengerdes RM, Roewe J, Mejias R, Marschall ALJ (2020). Applying antibodies inside cells: principles and recent advances in neurobiology, virology and oncology. BioDrugs.

[32] Lim EK, Kim T, Paik S, Haam S, Huh YM, Lee K (2015). Nanomaterials for theranostics: recent advances and future challenges. Chem Rev.

[33] Li H, Er Saw P, Song E (2020). Challenges and strategies for next-generation bispecific antibody-based anti-tumor therapeutics. Cell Mol Immunol.

[34] Gilad Y, Gellerman G, Lonard DM, O’Malley BW (2021). Drug combination in cancer treatment-from cocktails to conjugated combinations. Cancers (Basel).

[35] Huang R, Li X, He Y, Zhu W, Gao L, Liu Y (2020). Recent advances in CAR-T cell engineering. J Hematol Oncol.

[36] Liu Y, Sperling AS, Smith EL, Mooney DJ (2023). Optimizing the manufacturing and antitumour response of CAR T therapy. Nat Rev Bioeng.

[37] Page A, Hubert J, Fusil F, Cosset FL (2021). Exploiting B cell transfer for cancer therapy: engineered B cells to eradicate tumors. Int J Mol Sci.

[38] Nastasi C, Mannarino L, D’Incalci M (2020). DNA damage response and immune defense. Int J Mol Sci.

[39] Boi M, Gaudio E, Bonetti P, Kwee I, Bernasconi E, Tarantelli C (2015). The BET bromodomain inhibitor OTX015 affects pathogenetic pathways in preclinical B-cell tumor models and synergizes with targeted drugs. Clin Cancer Res.

[40] Zhang M, Zhang L, Hei R, Li X, Cai H, Wu X (2021). CDK inhibitors in cancer therapy, an overview of recent development. Am J Cancer Res.

[41] Whitfield JR, Soucek L (2025). MYC in cancer: from undruggable target to clinical trials. Nat Rev Drug Discov.

[42] Leskov I, Pallasch CP, Drake A, Iliopoulou BP, Souza A, Shen CH (2013). Rapid generation of human B-cell lymphomas via combined expression of MYC and Bcl2 and their use as a preclinical model for biological therapies. Oncogene.

[43] Ahmed EN, Cutmore LC, Marshall JF (2024). Syngeneic mouse models for pre-clinical evaluation of CAR T cells. Cancers (Basel).

[44] Vasan N, Baselga J, Hyman DM (2019). A view on drug resistance in cancer. Nature.

[45] Wallington-Beddoe CT, Mynott RL (2021). Prognostic and predictive biomarker developments in multiple myeloma. J Hematol Oncol.

[46] Lin P, Lin Y, Mai Z, Zheng Y, Zheng J, Zhou Z (2025). Targeting cancer with precision: strategical insights into TCR-engineered T cell therapies. Theranostics.

[47] Li X, Qi J, Wang J, Hu W, Zhou W, Wang Y (2024). Nanoparticle technology for mRNA: delivery strategy, clinical application and developmental landscape. Theranostics.

